# Multi‐omics revealed that DCP1A and SPDL1 determine embryogenesis defects in postovulatory ageing oocytes

**DOI:** 10.1111/cpr.13766

**Published:** 2024-12-04

**Authors:** Li Kong, Yutian Gong, Yongyong Wang, Mengjiao Yuan, Wenxiang Liu, Heyang Zhou, Xiangyue Meng, Xinru Guo, Yongbin Liu, Yang Zhou, Teng Zhang

**Affiliations:** ^1^ State Key Laboratory of Reproductive Regulation & Breeding of Grassland Livestock, College of Life Sciences Inner Mongolia University Hohhot China; ^2^ Department of Reproductive Medicine, Qingdao Municipal Hospital University of Health and Rehabilitation Sciences Qingdao China

## Abstract

Growing evidence indicates that the deterioration of egg quality caused by postovulatory ageing significantly hampers embryonic development. However, the molecular mechanisms by which postovulatory ageing leads to a decline in oocyte quality have not been fully characterized. In this study, we observed an accelerated decay of maternal mRNAs through RNA‐seq analyses in postovulatory‐aged (PostOA) oocytes. We noted that these downregulated mRNAs should be degraded during the 2‐cell stage. Proteomic analyses revealed that the degradation of maternal mRNAs is associated with the accumulation of DCP1A. The injection of exogenous *Dcp1a* mRNA or siRNA into MII stage oocytes proved that DCP1A could accelerate the degradation of maternal mRNAs. Additionally, we also found that SPDL1 is crucial for maintaining spindle/chromosome structure and chromosome euploidy in PostOA oocytes. *Spdl1‐*mRNA injection remarkably recovered the meiotic defects in PostOA oocytes. Collectively, our findings provide valuable insights into the molecular mechanisms underlying postovulatory ageing.

## Graphical abstract

Construct postovulatory ageing protein map to deeply analyse the dynamic changes of postovulatory ageing proteins. DCP1A accumulation accelerates maternal mRNA decay in postovulatory‐aged oocytes. SPDL1 is crucial for maintaining spindle/chromosome structure and chromosome euploidy in PostOA oocytes.

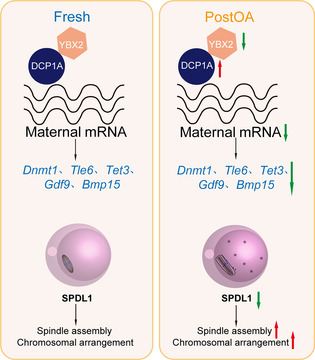

## 1 INTRODUCTION

Throughout recent decades, in vitro fertilization (IVF) is widely regarded as an effective treatment for infertility.^1^, ^2^ However, abnormal embryo development caused by the decline in oocyte quality in vitro has become one of the reasons that limit the development of human‐assisted reproductive technology (ART).^3^, ^4^ In mammals, there are two types of oocyte ageing: ‘maternal ageing’, caused by the ageing of the ovaries before ovulation, and ‘postovulatory ageing’, caused by the inability of the oocytes to fertilize at the optimal time after ovulation and their prolonged stagnation in the MII phase.^5^, ^6^ Furthermore, there is growing evidence that postovulatory‐aged (PostOA) oocyte quality declines in a time‐dependent manner^7^, ^8^ and is thought to be a key factor affecting fertilization and early embryo development.^9^, ^10^ Although the effects of postovulatory ageing have been extensively explored, the specific molecular mechanisms underlying the decline in oocyte quality remain poorly understood.

Maternal mRNAs are synthesized and stored during oocyte growth and remain in a constant state during the MII stage.^11^, ^12^ During the process of maternal‐zygotic transition (MZT), the maternal mRNA becomes extremely unstable and begins to degrade.^13^, ^14^ Interestingly, oocytes need to rely on the stored maternal mRNAs for oocyte maturation, fertilization, and early embryonic development.^15^, ^16^ Therefore, a growing number of reports have identified that maternal mRNA plays a pivotal role in oocyte maturation and post‐fertilization embryonic development.^17^, ^18^ Moreover, recent research shows that postovulatory ageing significantly degradation the maternal mRNAs, including *Nlrp5*, *Brg1*, *Mos* and *Zar1*.^5^, ^19^ Zygotic arrest 1 (ZAR1) with Y‐box binding protein 2 (MSY2) co‐regulates the stability of the maternal transcriptome, determining oocyte maturation and MZT.^14^ MSY2 plays a crucial role in regulating mRNA stability,^5^ dependent on downstream DCP1A and DCP2^12^, ^20^, ^21^ can lead to irreversible mRNA degradation, and the mobilization of *Cnot7* that causes mRNA deadenylation.^22^, ^23^, ^24^ However, the underlying mechanism of maintaining maternal mRNA stabilization after ovulation remains unknown.

In the process of oocyte meiotic maturation, the precise segregation of chromosomes is fundamental to the reproduction and development of organisms.^25^, ^26^ However, with prolonged periods of mature MII oocytes ageing in vitro, the risk of chromosomal abnormalities is increased, which further leads to neonatal developmental defects and embryonic death.^8^, ^27^ The function of spindle assembly checkpoint (SAC)‐associated proteins is to ensure accurate segregation of homologous chromosomes and sister chromatids.^28^ A series of fundamental findings in this field have been successively discovered. For instance, oocyte senescence affects the ability of chromosomes to segregate normally caused by a decrease in securin protein of the SAC‐anaphase‐promoting complex or cyclosome (SAC‐APC/C) axis,^29^ and PostOA oocytes lack mitotic arrest deficient 2 (MAD2) SAC protein, which results in premature centromere segregation and increased chromosomal aneuploidy during mitosis.^30^, ^31^ Thus, chromosomal aneuploidy due to impaired spindle assembly checkpoints is a key factor in the decline of oocyte quality after ovulation.

Improving the quality of oocytes ageing in vitro is fundamental to optimizing ART, especially when rescue intracytoplasmic sperm injection (ICSI) is used. In this study, proteomic and RNA sequencing analyses revealed that DCP1A accumulation causes accelerated decay of maternal mRNA and abnormal early embryonic development in PostOA oocytes. In addition, we found that postovulatory ageing causes oocyte spindle assembly disruption and increased sister chromosome mispairing, which can be alleviated by SPDL1. In conclusion, these findings are certainly useful for improving the quality of postovulatory aged oocytes and selecting candidate regulatory factors for further study.

## 2 RESULTS

### 2.1 Characterization of proteomic patterns in postovulatory‐aged oocytes

**FIGURE 1:**
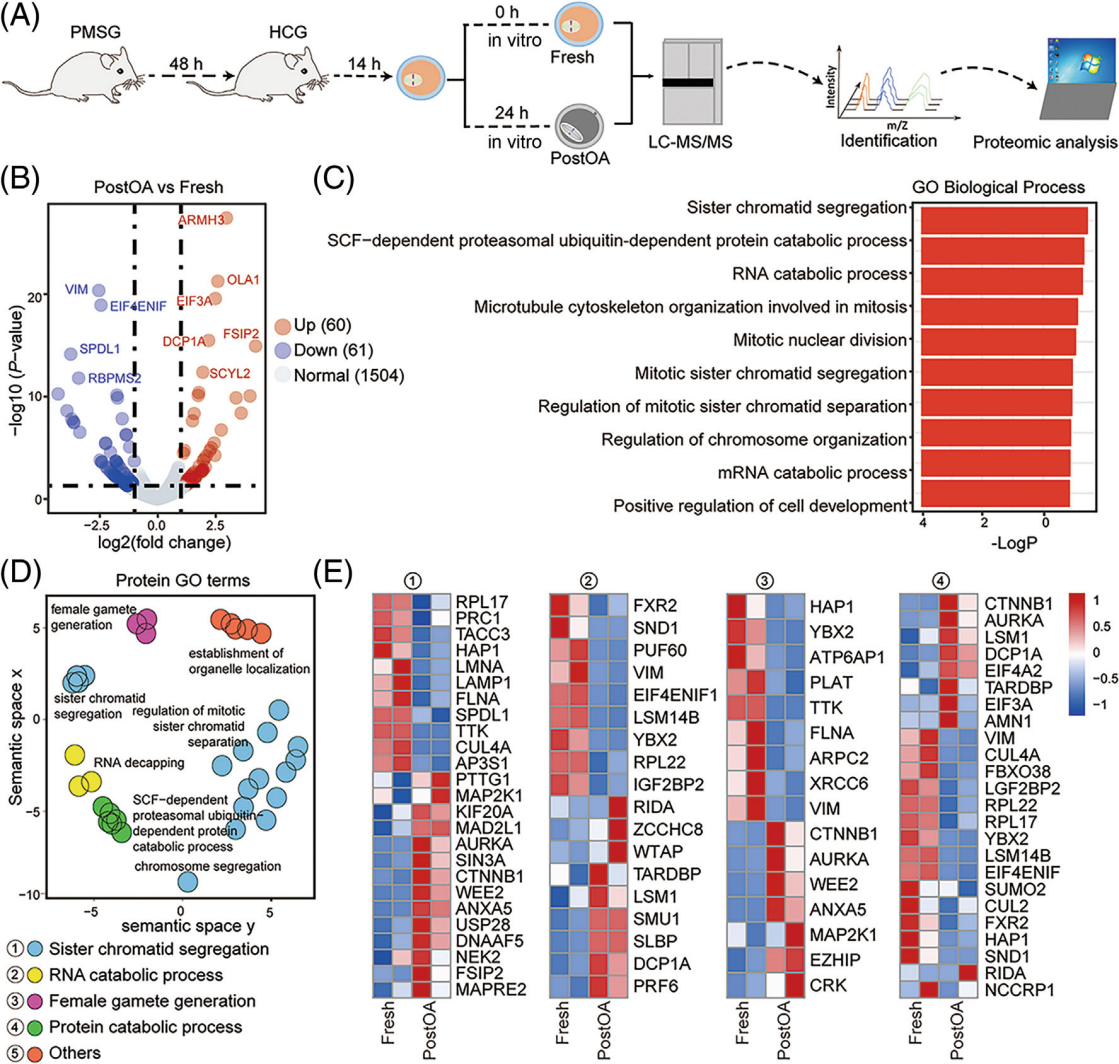
Proteomic analysis of postovulatory‐aged oocytes. (A) Illustration of metaphase II stage mouse oocytes isolated in vitro, and schematic overview of the workflow for proteomic profiling in oocytes. (B) Volcano plot showing the differentially expressed proteins (DEPs, *p*‐value < 0.05, downregulated, log2 fold change ≤ −1, blue; upregulated, log2 fold change ≥1, red; normal, grey) in mouse postovulatory aged (PostOA) oocytes compared with Fresh ones. Some highly DEPs are listed. (C) Gene ontology (GO) biology process (BP) enrichment analysis of DEPs in mouse PostOA oocytes compared with Fresh ones. (D) Hierarchical networks of the abundance of GO terms (Fisher's exact test, *p*‐value < 0.05) related to biological processes using REVIGO (Resnik measurement, 0.7 distance). The GO BP terms correspond to the DEPs shown in b. Reg., regulation. The images were generated using MediaLab. (E) Heatmaps of relative levels of the indicated proteins in ① sister chromatid segregation, ② RNA catabolic process, ③ female gamete generation, and ④ protein catabolic process pathways in mouse oocytes at PostOA and Fresh groups.

Previous studies have found that if ovulated oocytes cannot fertilize within the optimal time, the oocyte quality will deteriorate in a time‐dependent manner.^32^, ^33^ Although changes in postovulatory ageing in vitro have been reported,^34^, ^35^, ^36^ a comprehensive proteomic profile of postovulatory‐aged (PostOA) oocytes is lacking. Here, we performed proteomic analysis of ovulated oocytes from Fresh and PostOA groups (Figure 1A). About 2000 ovulated oocytes of each group (fresh and postovulatory‐aged) were collected and used for proteomic analysis.

A total of 9706 unique peptides were detected, and 1582 proteins were quantified (Figure S1A). Volcano plot and heatmap data reflected that the proteomic profile of PostOA oocytes was different from that of Fresh oocytes, showing that 61 differentially expressed proteins (DEPs) were downregulated, and 60 DEPs were upregulated in PostOA oocytes compared to Fresh oocytes (Figure 1B, Figure S1B, Table S1). Gene Ontology (GO) biology process (BP) analysis of DEPs showed a significant enrichment of categories related to sister chromatid segregation, and RNA catabolic process (Figure 1C). Next, we enrichment analysis of top50 GO BP terms found that sister chromatid separation, RNA catabolic process, female gamete generation, and protein catabolic processes were the main GO BP terms enrichment results (Figure 1D), and the specific protein level changes were represented as a heatmap (Figure 1E). For example, DEPs involved in chromatid separation, such as SPDL1 and TTK,^25^ might affect the spindle/chromosome structure; and DEPs involved in mRNA decapping, such as YBX2 and DCP1A, potentially influence the maternal mRNAs stabilization of ovulated oocyte.^37^, ^38^, ^39^ These proteins are implicated in the progression of oocyte meiosis and are essential for oocyte quality. Collectively, proteomic data provide a broad resource of postovulatory ageing and supply a practical entry point for exploring the mechanism of postovulatory ageing.

### 2.2 Accelerated decay of maternal mRNAs in postovulatory‐aged oocytes

**FIGURE 2:**
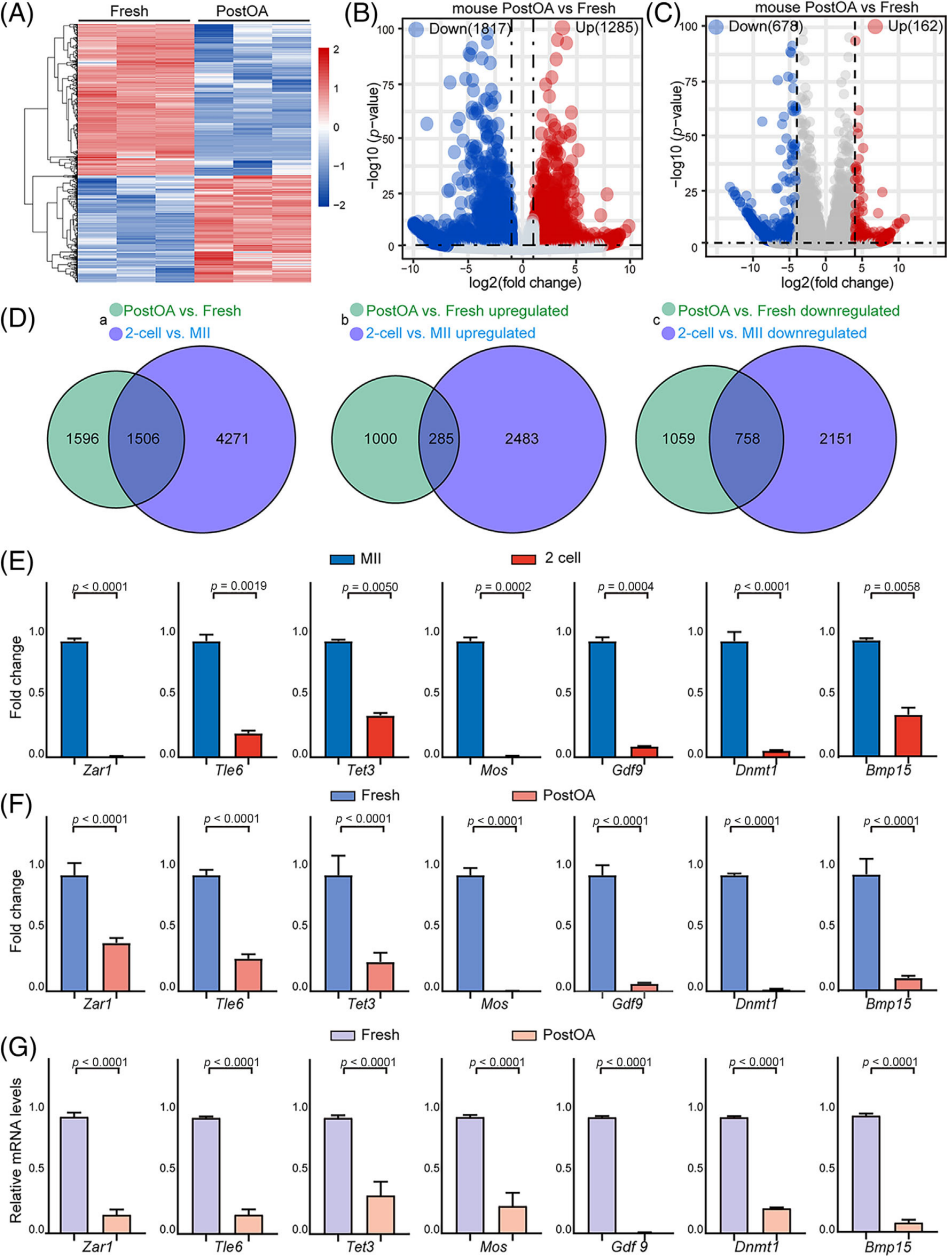
Transcriptome signature of postovulatory‐aged oocytes. (A) The heatmap demonstrates the differentially expressed genes (DEGs, 3102) in mouse oocytes at PostOA and Fresh oocytes. (B) Volcano plot showing the DEGs (3102) that were downregulated (blue; *p*‐value < 0.05, log2 fold change ≤ −1) and upregulated (red; *p*‐value < 0.05, log2 fold change ≥1) in mouse PostOA oocytes compared with Fresh ones. (C) Volcano plot showing the DEGs (840) that were downregulated (blue; *p*‐value < 0.05, log2 fold change ≤ −4) and upregulated (red; *p*‐value < 0.05, log2 fold change ≥4) in mouse PostOA oocytes compared with Fresh ones. (D) Veen diagram showing the overlap of (a) total transcripts from PostOA vs. Fresh; and 2‐cell vs. MII in mouse oocytes; (b) upregulated transcripts from PostOA vs. Fresh, and 2‐cell vs. MII in mouse oocytes, and (c) downregulated transcripts from PostOA vs. Fresh; and 2‐cell vs. MII in mouse oocytes. (E) RNA‐seq results showing the relative expression levels of representative genes in mouse MII and 2‐cell oocytes. Bars indicate the range of *p*‐value. (F) RNA‐seq results showing the relative expression levels of representative genes in mouse Fresh and PostOA oocytes. Bars indicate the range of *p*‐value. (G) RT‐PCR results showing the relative mRNA expression levels of representative genes in mouse PostOA oocytes compared with Fresh oocytes. *n* = 9 technical replicates. Error bars, mean ± SEM; by two‐tailed student's *t*‐test.

Given that proteomic analysis showed a significant enrichment of categories related to RNA catabolic process. We then performed transcriptome analysis of ovulated oocytes from Fresh and PostOA groups. Volcano plot and heatmap data show that compared with Fresh oocytes, 1285 and 1817 transcripts were increased or decreased by more than two‐fold changes in PostOA oocytes (Figure 2A,B, Table S2). In addition, 76 GO BP results of differentially expressed genes (DEGs) overlapped with those of DEPs, which were mainly enriched in GO BP terms such as ‘nuclear separation, chromatin separation, and mRNA processing’ (Figure S2A–D). Collectively, all of these results proved that the proteome and transcriptome can be mutually authentication, and provide further evidence that maternal mRNA stores decrease with ageing in vitro.

Interestingly, from transcriptome data analysis we found more down‐regulated DEGs than up‐regulated DEGs. Subsequently, this trend became even more remarkable when we increased the thresholds of the analyses, with 162 upregulated and 678 downregulated transcripts in PostOA oocytes (|log_2_(Fold change)| ≥ 4 and *p*‐value < 0.05) (Figure 2C). Multiple studies have shown that maternal mRNAs are degraded and cleared during the MZT,^37^, ^38^ whereas 90% of maternal mRNAs are degraded at the 2‐cell stage.^18^ Given that the transcripts were downregulated in PostOA oocytes, we further evaluated global mRNA levels based on the data of transcripts at the MII oocyte and 2‐cell stages.^39^ Comparison of the transcripts that were changed in PostOA/Fresh oocytes with those changed in 2‐cell/MII oocytes revealed high similarity between them (Figure 2Da). Particularly, many transcripts downregulated in PostOA/Fresh oocytes were also found to be decreased in 2‐cell/MII oocytes (Figure 2Dc). We further monitored the expression changes of important maternal mRNA, such as *Zar1*, *Tle6*, *Tet3, Mos, Gdf9, Bmp15* and *Dnmt1*.^14^, ^19^, ^40^, ^41^, ^42^ We found that these maternal mRNAs were significantly downregulated in the 2‐cell rather than in the MII oocyte stage (Figure 2E). However, our transcriptome results found that the transcript levels of important maternal mRNAs were also markedly reduced in PostOA oocytes compared with Fresh ones (Figure 2F). The changes in the expression of representative transcripts were validated by RT‐PCR (Figure 2G). These results demonstrate that maternal mRNA clearance may be triggered during postovulatory ageing.

### 2.3 RNA decapping involved in maternal mRNA decay in postovulatory‐aged oocytes

**FIGURE 3:**
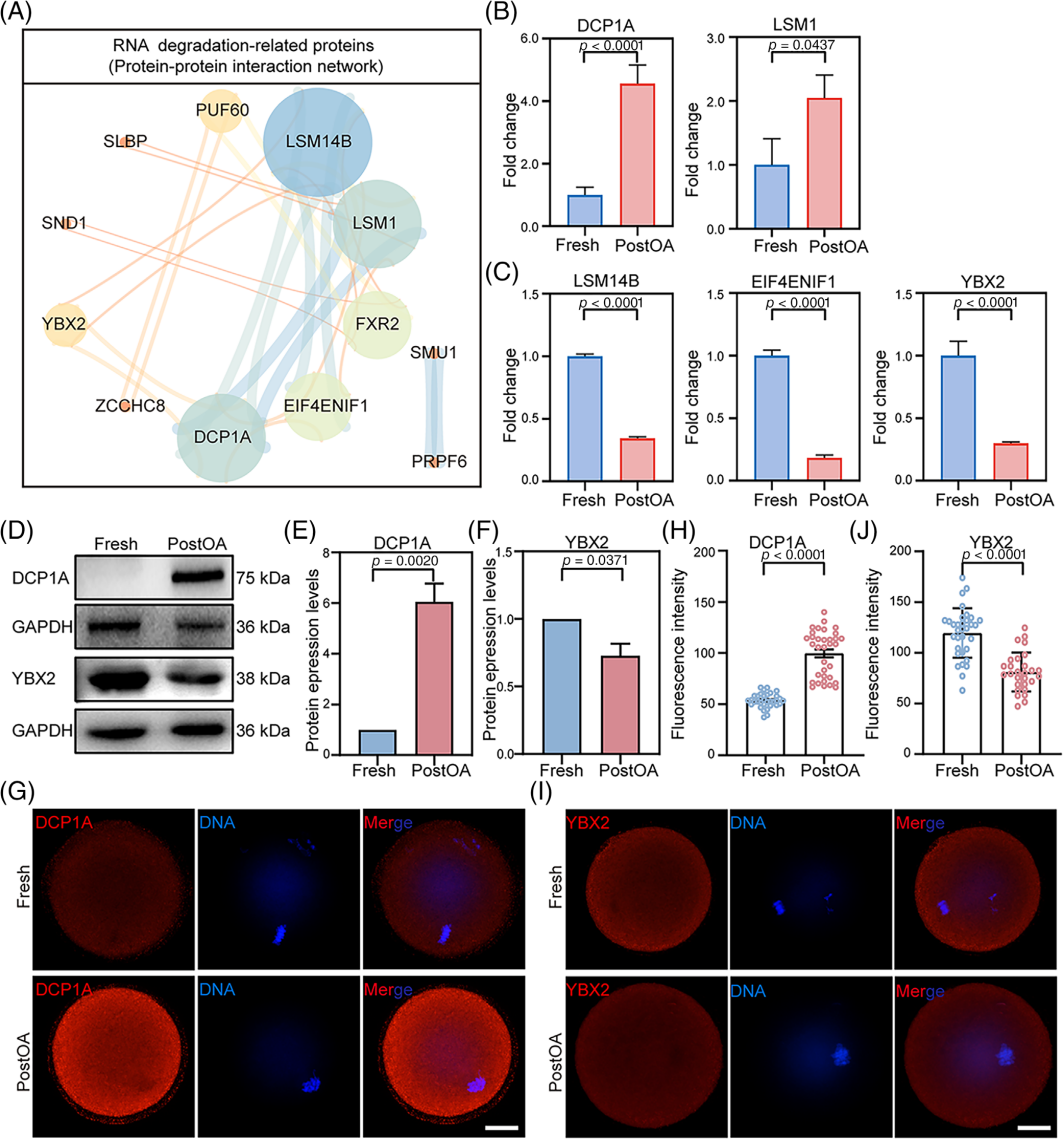
Postovulatory ageing affects the expression of RNA degradation‐related proteins. (A) Protein–protein interaction (PPI) network diagram corresponding to RNA degradation‐related proteins. (B, C) Proteomic results showing the 5′ cap relative expression levels of representative proteins in mouse PostOA oocytes compared with Fresh ones. (D) Western blot representative image of DCP1A and YBX2 in mouse PostOA oocytes compared with Fresh oocytes. Total proteins from 100 oocytes were loaded in each lane. GAPDH was blotted as a loading control. (E, F) Western blot results of DCP1A and YBX2 levels in mouse oocytes. *n* = 3 technical replicates. Error bars, mean ± SEM; by two‐tailed student's *t*‐test. (G) Representative immunofluorescence image of DCP1A in mouse PostOA oocytes compared with Fresh oocytes. Scale bar, 20 μm; red, DCP1A; blue, Hoechst. (H) Quantification of DCP1A immunofluorescence signals. Error bars, mean ± SEM; by two‐tailed student's *t*‐test. (I) Representative immunofluorescence image of YBX2 in mouse PostOA oocytes compared with Fresh oocytes. Scale bar, 20 μm; red, YBX2; blue, Hoechst. (J) Quantification of YBX2 immunofluorescence signals. Error bars, mean ± SEM; by two‐tailed Student's *t*‐test.

To unravel the potential molecular mechanisms underlying the clearance of maternal mRNAs in PostOA oocytes, we conducted a thorough analysis of the proteomic data associated with the RNA catabolic process during PostOA oocytes. In particular, protein–protein interaction (PPI) network analysis found that RNA degradation‐related proteins are more closely related to each other (Figure 3A). Interestingly, the proteins that mediate mRNAs 5′ decapping: such as mRNA‐decapping enzyme 1A (DCP1A)^21^ and sm‐like1 (LSM1)^43^, ^44^ were significantly increased (Figure 3B); mediate mRNA translation: such as Y‐box‐binding protein 2 (YBX2),^24^ LSM family member 14 (LSM14B),^45^, ^46^, ^47^, ^48^ and eukaryotic translation initiation factor 4E nuclear import factor 1 (EIF4ENIF)^49^, ^50^ were significantly down‐regulated (Figure 3C); but mediate mRNAs poly(A) tails shortened included: b‐cell translocation gene‐4 (BTG4),^51^ CCR4‐NOT transcription complex subunit 7 (CNOT7),^52^ eukaryotic translation initiation factor 4E (EIF4E), and eukaryotic translation initiation factor 4G1, 2 (EIF4G1, 2)^53^ were not significantly changed (Figure S3A). The findings were substantiated by performing immunofluorescence and western blot analysis of DCP1A and YBX2 (Figure 3D–J). These results provide evidence that postovulatory ageing induces the degradation of maternal mRNAs by facilitating RNA decapping.

### 2.4 Accumulation of DCP1A increases the extent of degradation of maternal mRNAs during postovulatory ageing

**FIGURE 4:**
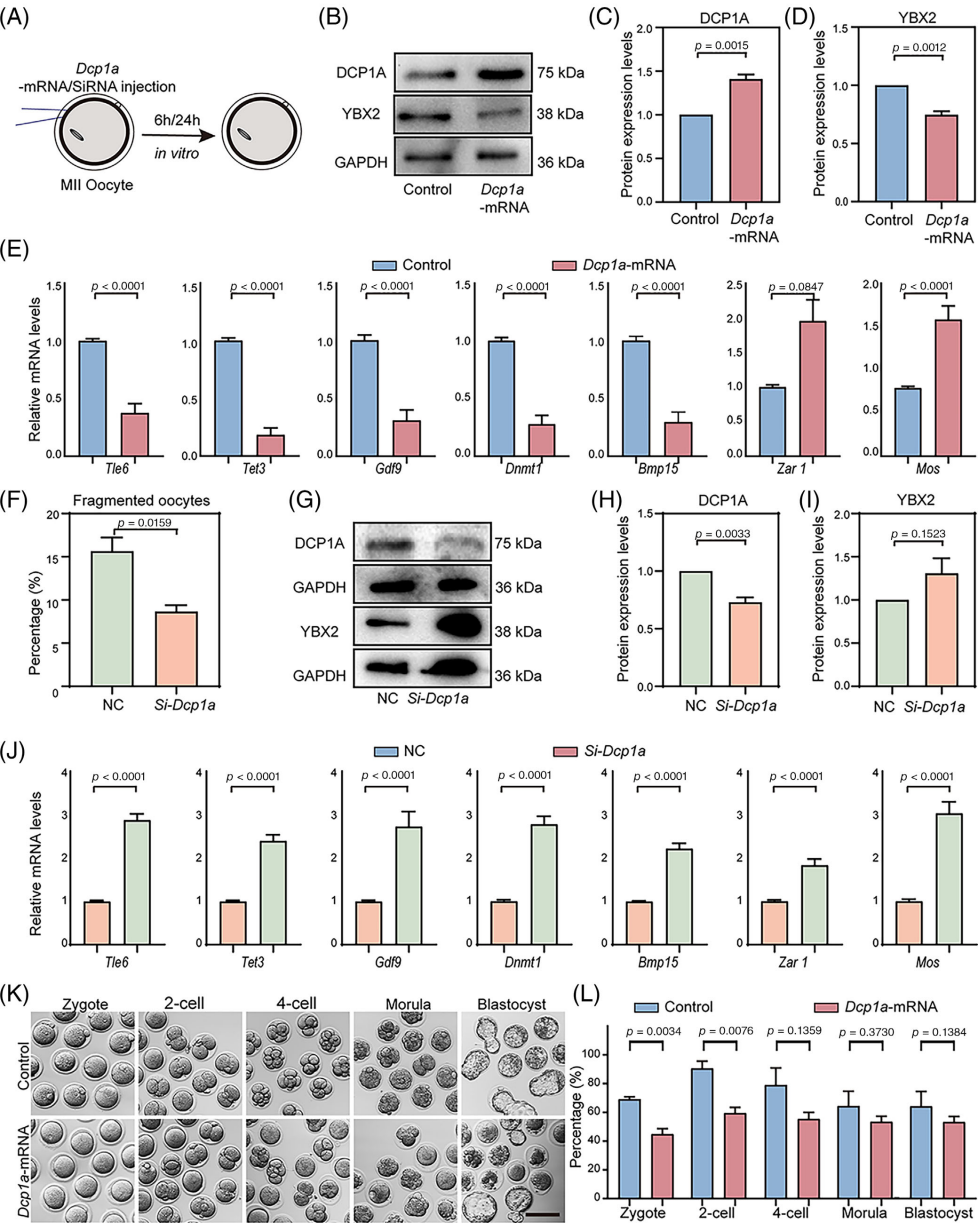
DCP1A plays an important role in the stabilization of maternal mRNA in postovulatory‐aged oocytes. (A) Schematic presentation of the DCP1A overexpression experiments. (B) Western blot representative image of DCP1A and YBX2 in mouse metaphase II oocytes DCP1A overexpression for 6 h. Total proteins from 100 oocytes were loaded in each lane. GAPDH was blotted as a loading control. (C, D) Western blot results of DCP1A and YBX2 levels in mouse metaphase II oocytes DCP1A overexpression for 6 h. *n* = 3 technical replicates. Error bars, mean ± SEM; by two‐tailed student's *t*‐test. (E) RT‐PCR results showing the relative mRNA expression levels of representative genes in mouse MII oocytes DCP1A overexpression for 6 h. *n* = 9 technical replicates. Error bars, mean ± SEM; by two‐tailed student's *t*‐test. (F) The rate of fragmentation was recorded in mouse metaphase II oocytes DCP1A knockdown for 24 h. *n* = 3 technical replicates. Error bars, mean ± SEM; by two‐tailed student's *t*‐test. (G) Western blot representative image of DCP1A and YBX2 in mouse metaphase II oocytes DCP1A knockdown for 24 h. Total proteins from 100 oocytes were loaded in each lane. GAPDH was blotted as a loading control. (H, I) Western blot results of DCP1A and YBX2 levels in mouse metaphase II oocytes DCP1A knockdown for 24 h. *n* = 3 technical replicates. Error bars, mean ± SEM; by two‐tailed student's *t*‐test. (J) RT‐PCR results showing the relative mRNA expression levels of representative genes in mouse MII oocytes DCP1A knockdown for 24 h. *n* ≥ 6 technical replicates. Error bars, mean ± SEM; by two‐tailed student's *t*‐test. (K) Representative images of early embryos developed from mouse metaphase II oocytes DCP1A overexpression for 6 h. Scale bar, 100 μm. (L) The rate of 2‐cell, 4‐cell, morula, and blastocyst embryos was recorded in the mouse metaphase II oocytes DCP1A overexpression for 6 h, respectively. *n* = 3 technical replicates. Error bars, mean ± SEM; by two‐tailed student's *t*‐test.

Previous experiments showed that DCP1A plays a crucial role in the degradation of mRNA during oocyte maturation in mice.^12^ Importantly, the influx of maternally recruited DCP1A is a crucial factor in triggering the transition of mRNA stability to instability during the process of meiotic maturation.^20^ Moreover, proteomic results showed that DCP1A exhibits a significantly elevated during postovulatory ageing (Figure S3B). To investigate whether mRNA degradation was accelerated by DCP1A, we microinjected mRNA and siRNA encoding DCP1A into MII oocytes. In vitro‐aged MII oocytes of *Dcp1a*‐mRNA or Control‐mRNA injection were collected after culture for 6 h (Figure 4A). Both western blot and immunofluorescence analyses showed that DCP1A was increased in oocytes of microinjected *Dcp1a*‐mRNA (Figure 4B,C, Figure S3C,D). Interestingly, our result found that DCP1A overexpression significantly reduced the YBX2 expression level (Figure 4B,D, Figure S3E,F); this was coordinated with the proteomes of PostOA oocytes. As expected, the stability of maternal mRNAs is also affected by DCP1A overexpression. RT‐PCR results revealed that the levels of maternal mRNAs, i.e., *Tle6*, *Tet3*, *Gdf9*, *Bmp15* and *Dnmt1* were significantly downregulated in DCP1A overexpressed oocytes (Figure 4E). *In vitro*‐aged MII oocytes of *Si‐Dcp1a* or NC injection were collected after culture for 24 h (Figure 4A). Upon DCP1A being knocked down, we found that the fragmentation rate of ageing oocytes was significantly reduced after ovulation (Figure 4F). Both western blot and immunofluorescence results showed that after microinjection of *si‐Dcp1a* into postovulatory aged oocytes, the expression level of DCP1A was significantly reduced, and the expression level of YBX2 tended to be increased (Figure 4G–I, Figure S3G–J). Not only that, RT‐PCR results found that DCP1A knockdown significantly increased the expression levels of key maternal mRNA that was reduced in postovulatory ageing (Figure 4J). These results suggest that recruited DCP1A is a key factor in maternal mRNA decay during postovulatory ageing due to decreased oocyte quality.

Previous work showed that maternal mRNA stabilization is tightly interconnected with the developmental potential of mammalian embryos. We found that postovulatory ageing dramatically reduced the capacity of fertilization and embryonic development (Figure S4A–F). We then investigated the fertilization capacity and embryonic developmental potential of DCP1A overexpressed oocytes. As expected, we observed that most control oocytes could be fertilized and developed into blastocysts, whereas DCP1A overexpressed oocytes had lower fertilization and 2‐cell rate compared with control oocytes (Figure 4K,L). These results demonstrated that DCP1A overexpression reduces the fertilization and embryonic development ability.

### 2.5 SPDL1 supplementation restores spindle/chromosome structure and euploidy in postovulatory‐aged oocytes

**FIGURE 5:**
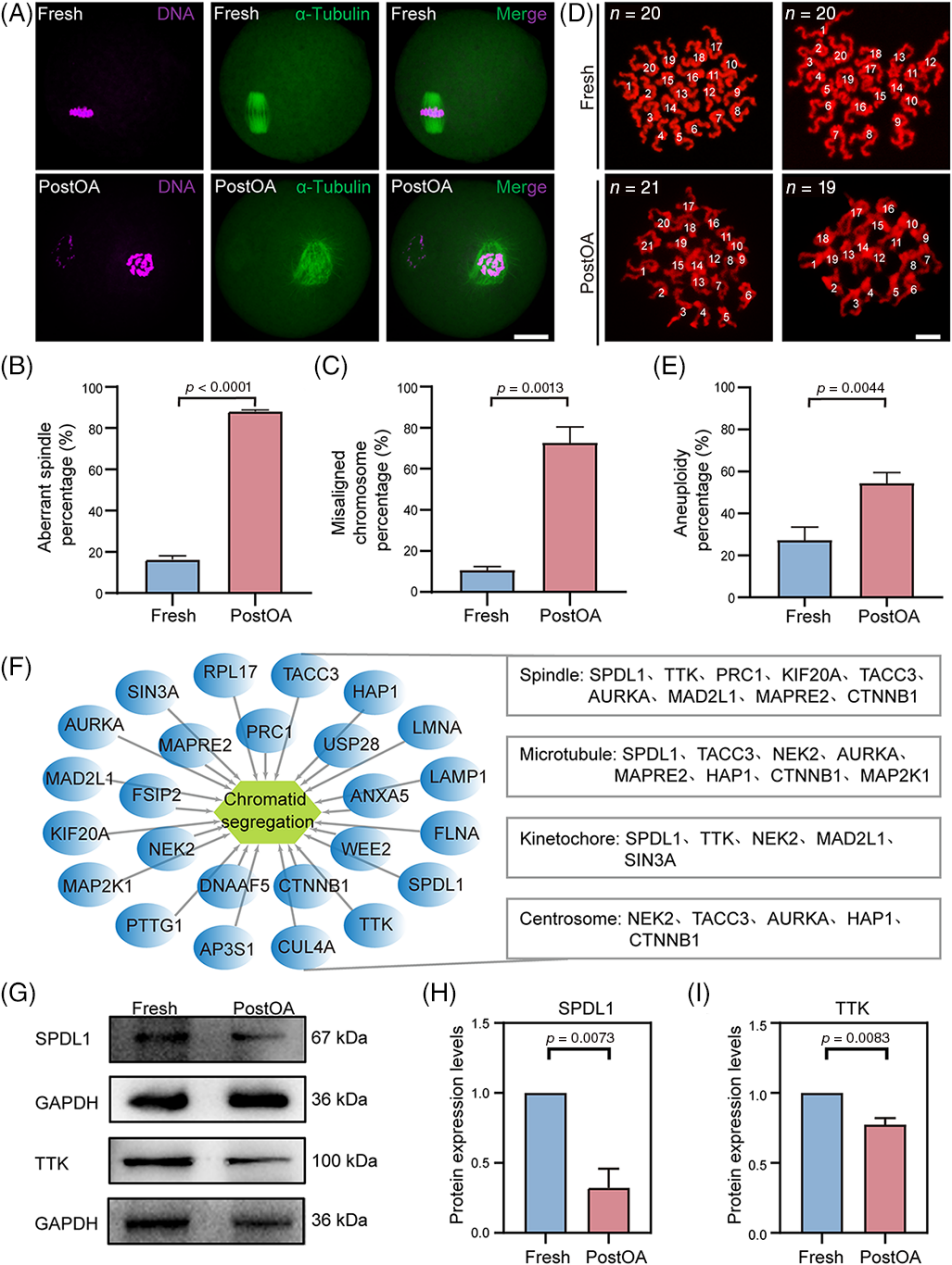
Postovulatory ageing affects spindle assembly and chromosomal euploidy. (A) Representative images of the spindle morphology and chromosome alignment at metaphase II oocytes. Oocytes were immunostained with the a‐tubulin‐FITC antibody to display the spindles and counterstained with Hoechst to visualize the chromosomes. Scale bars, 20 μm. Rose red, Hoechst; green, a‐tubulin‐FITC. (B) The rate of aberrant spindles at metaphase II was recorded in mouse PostOA oocytes compared with Fresh oocytes. *n* = 5 technical replicates. Error bars, mean ± SEM; by two‐tailed student's *t*‐test. (C) The rate of misaligned chromosomes was recorded in mouse PostOA oocytes compared with Fresh oocytes. *n* = 5 technical replicates. Error bars, mean ± SEM; by two‐tailed student's *t*‐test. (D) Representative images of euploid and aneuploid oocytes. Chromosome spreading was performed to count the chromosomes in mouse PostOA oocytes compared with Fresh oocytes. Scale bar, 5 μm. Red, Hoechst. (E) The rate of aneuploidy was recorded in mouse PostOA oocytes compared with Fresh oocytes. *n* = 3 technical replicates. Error bars, mean ± SEM; by two‐tailed student's *t*‐test. (F) Gene ontology network enrichment analysis of sister chromatid segregation. The spindle, microtubule, kinetochore, and centrosome functionally related proteins are shown in the boxes on the right. (G) Western blot representative image of SPDL1 and TTK in mouse PostOA oocytes compared with Fresh oocytes. Total proteins from 100 oocytes were loaded in each lane. GAPDH was blotted as a loading control. (H, I) Western blot results of SPDL1 and TTK levels in oocytes. *n* = 3 technical replicates. Error bars, mean ± SEM; by two‐tailed Student's *t*‐test.

Defects in spindle/chromosome structure are an important cause of failed meiosis and aneuploidy. A remarkably higher frequency of aberrant spindle/chromosome structure and aneuploid oocytes was found in PostOA oocytes compared with Fresh oocytes (Figure 5A–E). To explore the causes of aneuploidy in PostOA oocytes, sister chromatid segregation related proteins were analysed in proteomic data. The sister chromosome segregation related proteins were significantly changed in PostOA oocytes compared with Fresh oocytes (Figure S5A). Most of these DEPs are associated with spindle, microtubule, kinetochore, and centrosome (Figure 5F). Among them, studies have proven that spindle apparatus coiled‐coil protein 1 (SPDL1) is an important spindle checkpoint protein that plays a key role in the establishment of mitotic kinetochore‐microtubule attachments.^8^ Compared with Fresh oocytes in the proteome, SPDL1 (Fold change) was significantly downregulated in PostOA oocytes (Figure S5A). Moreover, the western blot results also showed that the expression of SPDL1 was decreased in PostOA oocytes compared with Fresh oocytes (Figure 5G,H). In addition, previous research found that mono‐polar spindle 1 (MPS1; also known as TTK) is an important regulator of meiosis and mainly functions in ensuring correct kinetochore‐microtubule attachments, which can cause conformational changes of spindle.^25^ The proteome and western blot results consistently showed that the expressions of TTK were significantly decreased (Figure 5G,I, Figure S5).

**FIGURE 6:**
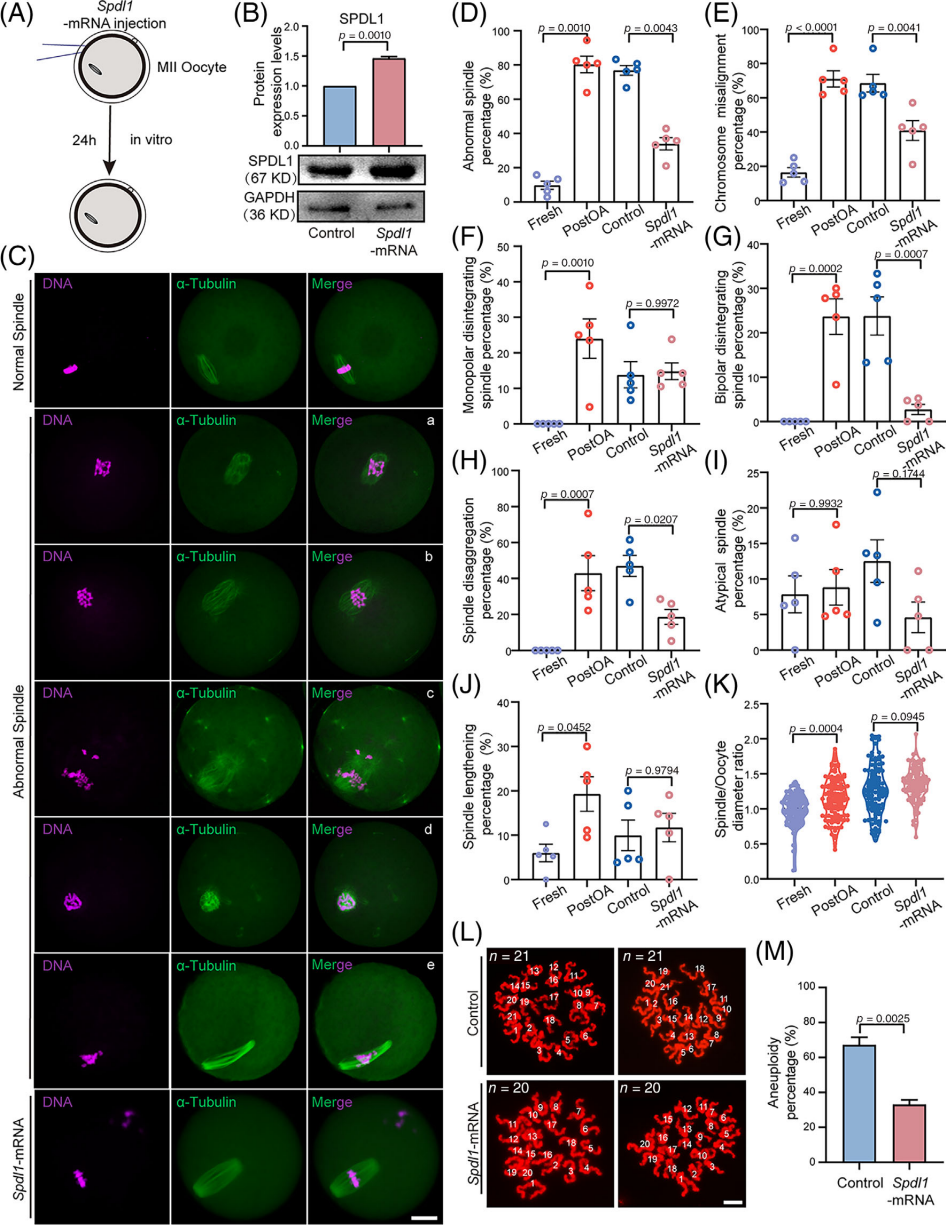
SPDL1 supplementation restores spindle/chromosome structure and euploidy in postovulatory‐aged oocytes. (A) Schematic presentation of the SPDL1 overexpression experiments. (B) Western blot representative image and result of SPDL1 in mouse metaphase II oocytes, SPDL1 overexpression for 24 h. Total proteins from 100 oocytes were loaded in each lane. GAPDH was blotted as a loading control. (C) Representative images of the spindle morphology and chromosome alignment of SPDL1 overexpression for 24 ho in mouse metaphase II oocytes. Scale bars, 20 μm. Rose red, Hoechst; green, a‐tubulin‐FITC. (D, E) The rate of total abnormal spindle/chromosome misalignment at metaphase II was recorded in mouse *Spdl1*‐mRNA compared with control‐mRNA oocytes. *n* = 5 technical replicates. Error bars, mean ± SEM; by two‐tailed student's *t*‐test. (F–J) The rate of (a) monopolar disintegration spindle, (b) bipolar disintegration spindle, (c) spindle disaggregation, (d) atypical spindle, and (e) spindle lengthening at metaphase II was recorded in mouse *Spdl1*‐mRNA compared with control oocytes, respectively. *n* = 5 technical replicates. Error bars, mean ± SEM; by two‐tailed student's *t*‐test. (K) The spindle/oocyte diameter ratio at metaphase II was recorded in mouse *Spdl1*‐mRNA compared with control‐mRNA oocytes. *n* = 5 technical replicates. Error bars, mean ± SEM; by two‐tailed student's *t*‐test. (L) Representative images of euploid and aneuploid oocytes. Chromosome spreading was performed to count the chromosomes in mouse *Spdl1*‐mRNA compared with control‐mRNA oocytes. Scale bar, 5 μm. Red, Hoechst. (M) The rate of aneuploidy was recorded in mouse *Spdl1*‐mRNA compared with control‐mRNA oocytes. *n* = 3 technical replicates. Error bars, mean ± SEM; by two‐tailed student's *t*‐test.

In brief, given that the SPDL1 was localized on kinetochore and spindle poles in MII oocytes.^54^ To explore the role of SPDL1 in PostOA oocytes, we expressed exogenous *Spdl1* in MII oocytes to monitor meiotic spindle/chromosome structure (Figure 6A). The SPDL1 protein level in Control‐mRNA or *Spdl1*‐mRNA injected oocytes after in vitro culture for 24 hours was confirmed by western blot (Figure 6B). We first classified the types of spindle defects and chromosome misalignment in PostOA oocytes. These defects include a variety of monopolar (a), apolar (b), depolymerized (c), atypical (d) and elongated (e) spindles with scattered (a, b, d) or lagging chromosomes (Figure 6Cc,e). Notably, spindle defects and failure of chromosome alignment in PostOA oocytes could be rescued by the expression of exogenous *Spdl1* (Figure 6D,E). Furthermore, we observed that apolar and depolymerized spindles were significantly reduced by SPDL1 overexpression (Figure 6G,H). However, we found that SPDL1 supplementation did not markedly recover the defects of monopolar, atypical and elongated spindles in PostOA oocytes (Figure 6F,I–K). As expected, SPDL1 overexpression significantly decreased the aneuploidy rate in the PostOA oocytes (Figure 6L,M). We conclude from these observations that SPDL1 is essential for the maintenance of spindle integrity by modulating spindle/chromosome structure during postovulatory ageing.

## 3 DISCUSSION

Postovulatory ageing has been shown to have a detrimental impact on both the quality and developmental capacity of oocytes. During postovulatory ageing, spindle defects,^8^ chromosomal aneuploidy^55^ and dynamics of mRNA^5^ have all been identified as significant factors leading to impaired oocyte quality. However, the exact molecular mechanism underlying these ageing‐induced defects remains largely unknown. In this study, we used an optimized LC–MS/MS method to systematically characterize the protein profile of mouse PostOA oocytes. We discovered notable protein enrichment features associated with maternal mRNA stability and spindle/chromosome structure.

Previous studies indicated that oocyte maturation, fertilization and zygotic genome activation (ZGA) are accompanied by maternal mRNA degradation,^17^, ^18^ and successful maternal mRNA clearance is associated with embryonic development in both mice and humans.^13^, ^38^ However, the dynamics of maternal mRNA decay in PostOA oocytes remain unclear. In this study, we found that RNA degradation‐related proteins are significantly enriched in PostOA oocytes. According to the previous findings, the transcript levels of PostOA oocytes altered, and it mainly reduced in the poly(A) tail length of maternal mRNAs,^5^, ^19^ especially the expression of *Tet3*, *Trim28*, *Zfp57*, *Dnmt1*, *Nlrp5*, *Nlrp14* and *Zar1*. Our transcriptomic results showed that the number of downregulated mRNAs significantly increased more than the upregulated mRNAs in PostOA oocytes. Furthermore, we observed that these downregulated mRNAs should be degraded during the 2‐cell stage. These data provide evidence for accelerated mRNA degradation during postovulatory ageing.

It is demonstrated that in eukaryotic cells maternal mRNA degradation involves distinct stages: CCR4‐NOT adenosylase‐dependent shortening of the 3′ poly(A) tail^56^ and decapping of the 5′ end triggered after resumption of meiotic oocyte division.^12^ According to the research shows that the MZT licensing factor BTG4 accelerates maternal mRNA degradation by recruiting the CCR4‐NOT deadenylase complex before fertilization.^39^ Interestingly, combined with our proteomic analysis, we found that BTG4 and CCR4‐NOT complex related proteins did not alter significantly in PostOA oocytes, which indicated that they did not play an essential role in the accelerated degradation of maternal mRNAs in PostOA oocytes. In addition, we found that the proteins of DCP1A and LSM1 were greatly upregulated, whereas YBX2 and LSM14B were significantly downregulated. DCP1A and DCP2 stimulate decapping activity and thereby promote removal of the cap structure at the 5′ end of the mRNA.^57^ Furthermore, the LSM1‐7 proteins form a stable complex with the decapping enzyme DCP1A^58^, ^59^ and the exonuclease XRN1P^60^ to promote the degradation of mRNA from 5′‐3′. Importantly, DCP1A is an important protein responsible for mRNA decapping. Recent studies have shown that cordycepin can inhibit the degradation of maternal mRNA by inhibiting the polyadenylation of DCP1A mRNA, thus preventing postovulatory ageing of mammalian oocytes and thereby promoting oocyte developmental capacity.^61^ Therefore, we speculated that the accumulation of DCP1A accelerates the abnormal degradation of maternal mRNAs in PostOA oocytes. Maternal mRNA instability and deregulation not only impair oocyte maturation but also decrease fertilization and embryonic development in PostOA oocytes.^13^, ^17^, ^18^ Through short‐term overexpression and knockdown experiments of DCP1A, we demonstrated that the accumulation of DCP1A caused by postovulatory ageing will destroy the stability of maternal mRNA, hinder early embryonic development, and significantly reduce the fertilization rate and 2‐cell rate of oocytes.

Maternal mRNA clearance defects can result in abnormalities in ZGA and early embryonic development.^14^, ^39^ Previous studies have shown that aged human and mouse oocytes display deficiencies in mRNA clearance during meiotic maturation, impacting early embryonic development.^62^, ^63^ This deficiency is linked to a lack of CXXC1, leading to reduced levels of H3K4me3 and hindering the degradation of maternal mRNA. Additionally, the research found that older women's fully‐grown oocytes were inefficient translation of transcripts encoding essential factors for maternal mRNA degradation.^62^ Our study unveiled that the accumulation of DCPIA in postovulatory aged oocytes accelerates the degradation of maternal mRNA, affecting early embryonic development. Remarkably, the consequences of the dynamic changes in two distinct maternal mRNA types manifest as defects in early embryonic development. The varying protein profiles in oocytes with different ageing patterns are identified as the primary factor contributing to this phenomenon. Nevertheless, further exploration is needed to understand the regulatory mechanisms underlying these differences in protein profiles.

The spindle/chromosome structure is crucial for the euploidy of chromosomes.^64^, ^65^ In this study, we identified the alterations of the spindle assembly and sister chromosome segregation related proteins in PostOA oocytes, of which SPDL1 expression was particularly reduced. SPDL1 is known for the recruitment of dynein to kinetochores in mitosis, which can control microtubule polarity.^66^ Depletion of SPDL1 causes mitosis metaphase arrest in different species.^66^, ^67^ Recent evidence discovered that SPDL1 as a spindle assembly checkpoint protein is essential for meiotic maturation.^54^ Based on this research, we suggested whether overexpression of SPDL1 at MII oocytes reduces abnormal spindle defects and chromosomal aneuploidy in PostOA oocytes, and the results showed that the spindle defects were partially rescued, especially the microtubule depolymerization is reduced. These findings suggest that SPDL1 plays an important role in regulating spindle microtubule integrity and chromosome alignment in PostOA oocytes, but it is still needed to explore the mechanism that cooperates with other spindle checkpoint proteins.

Our study has revealed that the accumulation of DCP1A in MII oocytes undergoing postovulatory ageing leads to the degradation of maternal mRNAs. Furthermore, we have identified the critical role of SPDL1 in maintaining the spindle morphology and chromosomal alignment of PostOA oocytes. In summary, this study generated a proteome profile that demonstrated RNA degradation and chromosomal aneuploidy in PostOA oocytes were important causes of oocyte quality decline.

## 4 MATERIALS AND METHODS

### 4.1 Mice

All mice used in this experiment were ICR, obtained from the Beijing Vital River Laboratory Animal Technology Co., Ltd., China. The mice were kept in the experimental animal room of Inner Mongolia University and were maintained at 20–22°C for 12 h of light and 12 h of dark every day, 50%–70% humidity and food and water provided at libitum. All experimental protocols involving mice were approved by the Inner Mongolia University Research Committee, and the rearing of mice was carried out following relevant policies and requirements.

### 4.2 Oocyte collection and in vitro culture

8‐weeks‐old ICR female mice were injected intraperitoneally with 10 IU of pregnant mare serum gonadotropin (PMSG, 54454, Ningbo) and 10 IU of human chorionic gonadotropin (HCG, 11008; Ningbo) after PMSG 48 hours later, and the mice were euthanized after HCG 14 h later. To collect MII oocytes, cumulus‐oocyte complexes (COCs) were obtained from the ampulla, then the oocytes and granulosa cells were separated by hyaluronidase (H4272; Sigma). MII oocytes were washed and incubated in M2 medium (M7167; Sigma) overlaid with mineral oil for 24 h in a 5% CO_2_ atmospheric at 37°C incubator.

### 4.3 Immunofluorescence

Collected oocytes were fixed in 4% paraformaldehyde in phosphate‐buffered saline (PBS) for 30 min and permeabilized in Triton X‐100 (9036; Sigma) for 20 min. After blocking with 1% bovine serum albumin (BSA, 4240GR025; BioFROXX) in PBS, oocytes were incubated with primary antibodies (Table S3) at 4°C overnight. Then sequentially labelled with secondary antibodies (Table S3) for 1 h at room temperature, and Hoechst 33342 (C1028; Beyotime) for 15 min. Next, the oocytes were imaged using a Zeiss LSM710 confocal microscope.

For the measurement of fluorescence intensity, ImageJ software was used to define a region of interest (ROI) from both control and treatment oocytes on the same glass slide and the same parameters with the microscope. The independent values of all oocytes were used to measure the fluorescence intensity.

### 4.4 Chromosome spreading

Collected oocytes were left in tyrode's buffer (pH 2.5, T1788; Sigma) for 5–10 s to remove the zona pellucida. Then immediately transfer the oocytes to M2 medium for 10 min and fix on glass slides with 1% paraformaldehyde, 0.15% TritonX‐100 and 3 mM dithiothreitol for 2 h at room temperature. After air‐drying, chromosomes were dyed with Hoechst 33342 and observed with the confocal microscope.

### 4.5 Western blot analysis

A total of 100 oocytes were used to denature the protein by 2 × loading buffer SDS (abs9237; Absin) and heated at 105°C for 10 min. Then the proteins were separated by SDS‐polyacrylamide gel electrophoresis (SDS‐PAGE) and transferred to polyethylene fluoride (PVDF) membranes (3010040001; Millipore). The following membranes were blocked in tris‐buffered saline and Tween 20 (TBST) containing 5% BSA for 2 h at room temperature. After incubating with primary antibodies (Table S3) at 4°C overnight, membranes were washed with TBST. Next, the membranes were incubated with HRP‐related secondary antibodies (Table S3) for 1.5 h at room temperature and washed with TBST three times. Finally, the membranes were coloured by BeyoECL plus kit (34095; ThermoFisher) and exposed using chemiluminescence according to the manufacturer's instructions.

### 4.6 In vitro fertilization and embryo culture

For the in vitro fertilization (IVF) procedure, the sperm of 8–10‐weeks‐old male was pre‐equilibrated in TYH (M2050; EasyCheck) medium at 37°C and 5% CO_2_ incubator for 1 h. Afterwards, the MII oocytes were transferred to human tubal fluid (HTF, M1130; EasyCheck) medium, which was equilibrated in advance, and added the spermatozoa approximate concentration 2.5 × 10^6^ sperms to HTF medium at 37°C and in 5% CO_2_ incubator for 4–6 h. The presence of two pronuclei was scored as successful fertilization. Then the embryos were cultured in a 3.5 cm culture dish containing KSOM (M1450; EasyCheck) under mineral oil at 37°C and in a 5% CO_2_ incubator for 4.5 days to obtain blastosphere.

### 4.7 Overexpression or knockdown analysis and microinjection

Total RNA was extracted from 100 oocytes using the RNeasy Micro Kit (74,004, Qiagen). After EasyScript one‐step gDNA removal and cDNA synthesis supermix (AL21115A, TAKARA), the mRNAs were reverse transcribed into cDNA, then the target gene was purified by PCR, and then cloned target genes into pcDNA3.1+ vector. Furthermore, use the Pure Yield Plasmid Miniprep System (A1223; Promega) plasmid extraction kit to extract the plasmid from *Escherichia coli* containing the target gene plasmid. Afterwards, the extracted plasmids were linearized with FastDigest ScaI (FD0434; Thermo Scientific) restriction enzyme. The digested linearized plasmid was subjected to agarose gel electrophoresis, the target band was cut out after exposure to the gel, and the plasmid was recovered using the Wizard® SV Gel and PCR Clean‐Up System (A9281; Promega) gel recovery kit. Subsequently, mRNAs were generated by in vitro transcription using the mMESSAGE mMACHINE® T7 Ultra Kit (AM1345; Invitrogen), and in vitro, transcribed mRNAs were purified using the RNAeasy MinElute Cleanup Kit (74004; Qiagen). Synthetic mRNAs were aliquoted and stored at −80°C. The siRNA of DCP1A was purchased from GenePharma (Suzhou). The relevant primer sequences are found in Table S4.

Microinjection of mRNA or siRNA was used to overexpress and knockdown the corresponding protein in mice oocytes. Microinjection needles and fixed needles were prepared by using a needle‐pulling instrument and a needle‐calcining instrument, and all oocytes were injected using micromanipulation equipment (Nicon‐Narishige). The mRNA concentration after in vitro transcription and purification was controlled at 150 ng/μL, and each oocyte was injected with about 10 pL. After all, injections were completed, the oocytes in good condition were placed in M2 medium under mineral oil at 37°C and in a 5% CO_2_ incubator culture.

### 4.8 RNA isolation and quantitative real‐time PCR

Total mRNAs were obtained from 100 oocytes by RNeasy Micro Kit according to the manufacturer's instructions. Then RNA was reversed into cDNA by EasyScript one‐step gDNA removal and cDNA Synthesis Supermix Kit and performed with SYBR Green Premix Ex Taq II (RR820A, TAKARA) on a Light Cycler 480II System (Roche Diagnostics) RT‐RCR system. Relative mRNAs expression levels were compared by the levels of endogenous *Gapdh* mRNA (internal control) and analysed by the difference multiple = 2^(−ΔΔCt)^ method. The primer sequences are listed in Table S4.

### 4.9 Proteomics

For each group of Fresh and PostOA, 2000 MII oocytes were obtained from ICR mice. Oocytes were lysed in SDT buffer to extract proteins, and proteins were quantified using a BCA protein assay kit (Bio‐Rad, USA). Then proteins were trypsinized to obtain peptides according to the filtration‐assisted sample preparation procedure described by Matthias Mann. The obtained peptides were coupled to a Q Exactive mass spectrometer (Thermo Scientific) and analysed using the positive ion mode of LC–MS/MS and run in mode with peptide identification enabled. Next, MS raw data for each sample were combined and searched for identification and quantification by using MaxQuant 1.5.3.17 software. For MII oocytes, protein sequences were searched using InterProScan software to identify protein domain signatures from the InterPro member database pfam. The protein sequences of the selected differentially expressed proteins were then subjected to a local search to find homologous sequences by NCBI BLAST+ client software (ncbi‐blast‐2.2.28+‐win32.exe) and InterProScan. Then differentially expressed proteins (DEGs) obtained with *p*‐value < 0.05 (by significant A/B^68^), and fold change >2 or fold change <0.5 were used for functional enrichment analysis. The proteomic data in this work have been deposited in the iProX under the ProteomeXchange ID accession number: PXD038293.

### 4.10 RNA‐seq library construction and data analysis

MII oocytes were collected from ICR mice (100 oocytes per sample). Total mRNAs were obtained from each group by RNeasy Mini kit according to the manufacturer's instructions. In the first step, Illumina was used for sequencing the library with RNA. In brief, mRNAs were fragmented by oligo (dT), then reverse transcribed to cDNA, and then PCR amplified to obtain the library with paired ends of 150 bp (PE150). We used the obtained raw lower sequences (Raw Reads) to remove low‐quality sequences and linker contamination by fastp to obtain high‐quality sequences (Clean Reads), and all subsequent analyses were based on clean reads. The clean reads were aligned with the reference genome with STAR, and finally, the readcounts were recalculated with feature counts. Differentially expressed genes (DEGs) were obtained using the R software DESeq2 package at a *p*‐value < 0.05, FDR < 0.05, and were used for functional enrichment analysis. The RNA‐seq data in this work have been deposited in the genome sequence archive (GSA) under accession number: CRA008757. The RNA‐seq data of MII and 2‐cell stage were obtained from NCBI Gene Expression Omnibus (GEO) accession number: GSE135787.

### 4.11 Statistical analysis

All results in this experiment are given as mean ± SEM. Most experiments included were repeated at least three independent times. Experimental groups were shown by GraphPad Prism 8.0 software and compared by two‐tailed unfixed Student's *t*‐test or one‐way analysis of variance (ANOVA). *p*‐value ≤ 0.05 was statistically significant difference and *p*‐value ≤ 0.01 was highly significant difference.

## AUTHOR CONTRIBUTIONS

Yang Zhou and Teng Zhang conceived and designed the experiments. Li Kong, Yutian Gong, Yongyong Wang, Mengjiao Yuan, Wenxiang Liu, Heyang Zhou, Xiangyue Meng, and Xinru Guo performed the experiments. Li Kong, Yongbin Liu, Yang Zhou, and Teng Zhang wrote and edited the manuscript. All the authors read the manuscript and approved the final manuscript.

## FUNDING INFORMATION

This study was supported by the Inner Mongolia Autonomous Region Open Competition Projects (2022JBGS0024 to TZ and YBL), the National Natural Science Foundation of China (32260180 to TZ; 32260181 to YZ), the Inner Mongolia Autonomous Region Science and Technology Plan (2023YFHH0114 to TZ), the Young Talents of Science and Technology in Universities of Inner Mongolia Autonomous Region (NJYT23013 to YZ), the Central Government Guides Local Science and Technology Development Fund Projects (2022ZY0188 to TZ), and the Science and Technology Major Project of Inner Mongolia Autonomous Region of China to the State Key Laboratory of Reproductive Regulation and Breeding of Grassland Livestock (2021ZD0048 to TZ and YZ).

## CONFLICT OF INTEREST STATEMENT

The authors declare that they have no known competing financial interests or personal relationships that could have appeared to influence the work reported in this paper.

## Supporting information


Figure S1

Table S1

Table S2

Table S3

## Data Availability

The accession number of mouse RNA sequencing data reported in this paper is in the GSA under accession number: CRA008757. The mass spectrometry proteomics data have been deposited to the ProteomeXchange Consortium (http://proteomecentral.proteomexchange.org) via the iProX partner repository^69^, ^70^ with the dataset identifier PXD038293.
