# Supplementary material for: Multi‐omics revealed that DCP1A and SPDL1 determine embryogenesis defects in postovulatory ageing oocytes
Source: Cell Prolif. 2024 Dec 4;58(3):e13766. doi: 10.1111/cpr.13766 (PMC11882766; doi:10.1111/cpr.13766)

**Figure Legend**

**Figure S1 Proteome profiling of postovulatory-aged oocytes.**

1. Identification of peptide numbers and protein results from proteomic samples.
2. The heatmap of relative levels of DEPs (121) in mouse PostOA and Fresh oocytes.

**Figure S2 Transcriptome profiling of postovulatory-aged oocytes.**

1. Gene ontology biological process enrichment analysis of DEGs in mouse PostOA oocytes compared with Fresh oocytes.
2. Veen diagram showing the overlap of proteomic GO-BP terms with transcriptome GO-BP terms in mouse Fresh oocytes compared with PostOA oocytes.
3. Gene ontology biological process enrichment analysis of top20 transcriptome co-GO-BP terms from proteomic and transcriptome.
4. Gene ontology biological process enrichment analysis of top20 proteomic co-GO-BP terms from proteomic and transcriptome.

**Figure S3 Expression levels of RNA catabolic process proteins in postovulatory-aged oocytes.**

1. Proteomic results showing the 3’ poly(A) tails relative expression levels of representative proteomic in mouse PostOA oocytes compared with Fresh ones.
2. Proteomic results showing the relative expression levels of RNA catabolic process representative proteins in mouse PostOA oocytes compared with Fresh ones.
3. Representative immunofluorescence image of DCP1A in DCP1A overexpression oocytes compared with control oocytes. Scale bar, 20 μm. Green, DCP1A; blue, Hoechst.
4. Quantification of DCP1A immunofluorescence signals. Error bars, mean ± SEM; by two-tailed Student’s t-test.
5. Representative immunofluorescence image of YBX2 in DCP1A overexpression oocytes compared with control oocytes. Scale bar, 20 μm. Red, YBX2; blue, Hoechst.
6. Quantification of YBX2 immunofluorescence signals. Error bars, mean ± SEM; by two-tailed Student’s t-test.
7. Representative immunofluorescence image of DCP1A in DCP1A knockdown oocytes compared with control oocytes. Scale bar, 20 μm. Red, DCP1A; blue, Hoechst.
8. Quantification of DCP1A immunofluorescence signals. Error bars, mean ± SEM; by two-tailed Student’s t-test.
9. Representative immunofluorescence image of YBX2 in DCP1A knockdown oocytes compared with control oocytes. Scale bar, 20 μm. Red, YBX2; blue, Hoechst.
10. Quantification of YBX2 immunofluorescence signals. Error bars, mean ± SEM; by two-tailed Student’s t-test.

**Figure S4 Effects of postovulatory aging on oocyte fertilization ability and early embryo developmental potential.**

1. Representative images of early embryos developed from oocytes. Scale bar, 100 μm.
2. The rate of zygote embryos was recorded in the Fresh and PostOA groups. n = 5 technical replicates. Error bars, mean ± SEM; by two-tailed student’s t-test.

(C-F) The rate of 2-cell, 4-cell, embryos morula, and blastocyst embryos was recorded in the Fresh, and PostOA groups. n = 5 technical replicates. Error bars, mean ± SEM; by two-tailed student’s t-test.

**Figure S5 Expression levels of sister chromatid-associated proteins in postovulatory-aged oocytes.**

1. Proteomic results showing the relative expression levels of sister chromosome segregation representative proteins in mouse PostOA oocytes compared with Fresh ones.


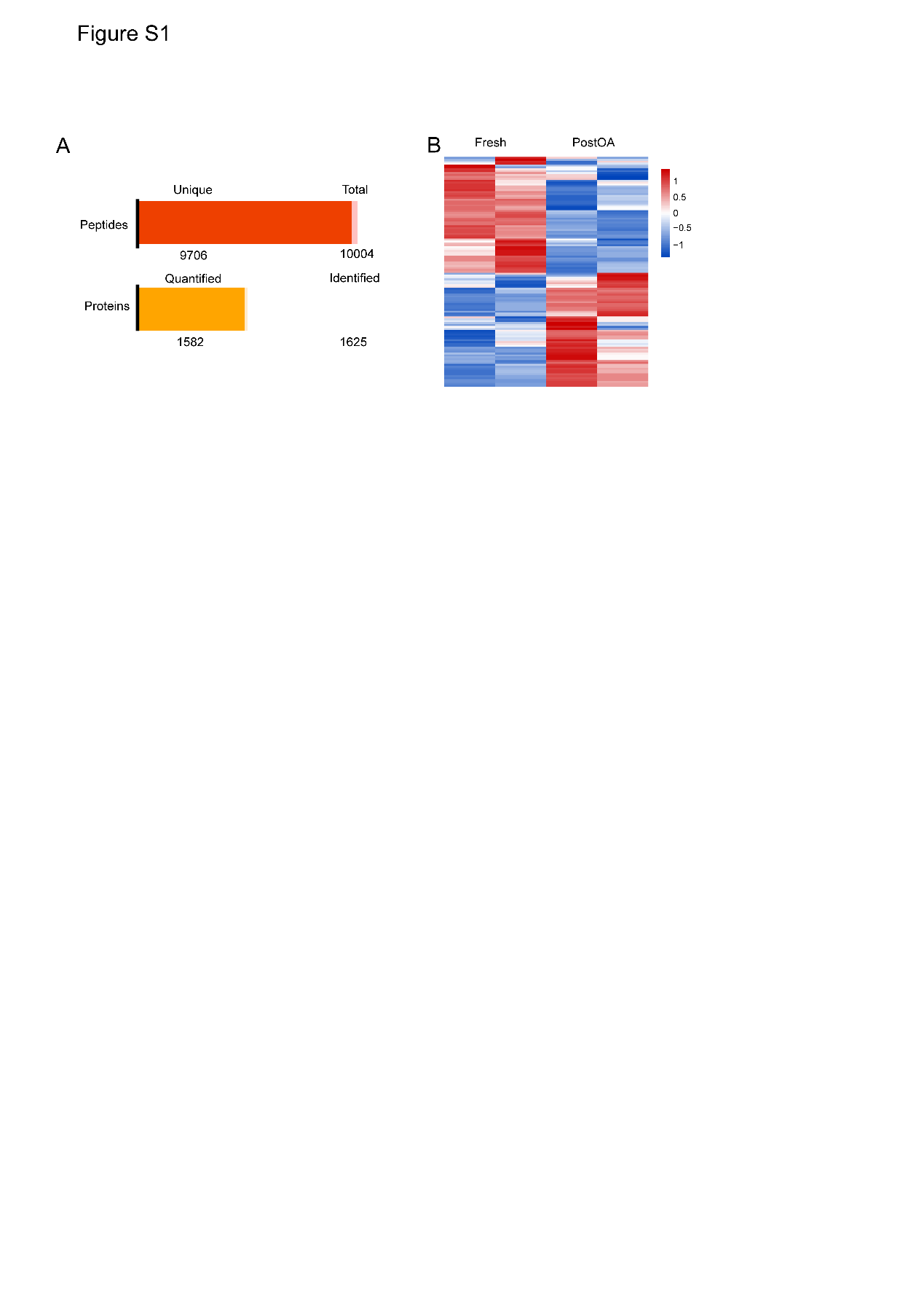


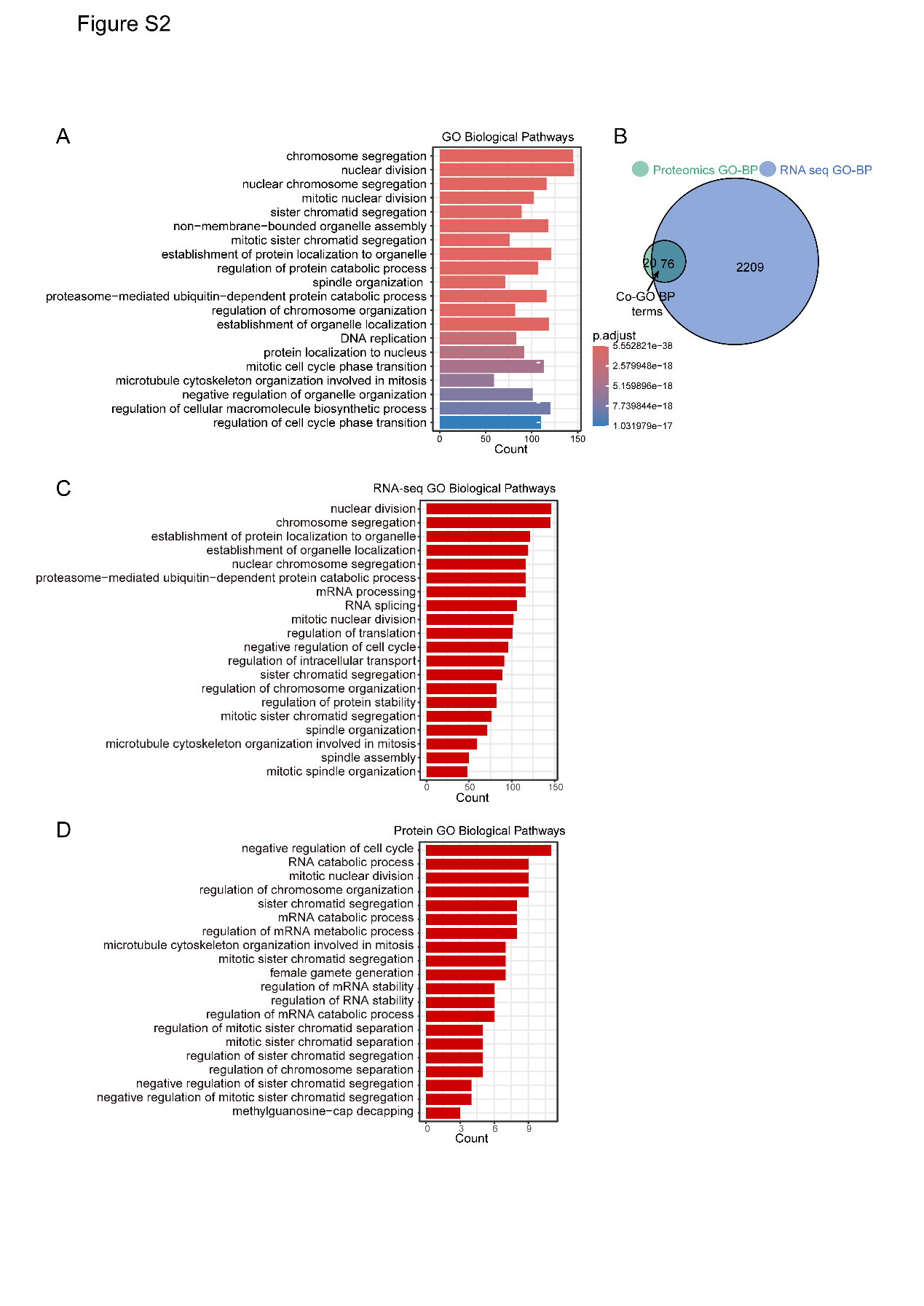


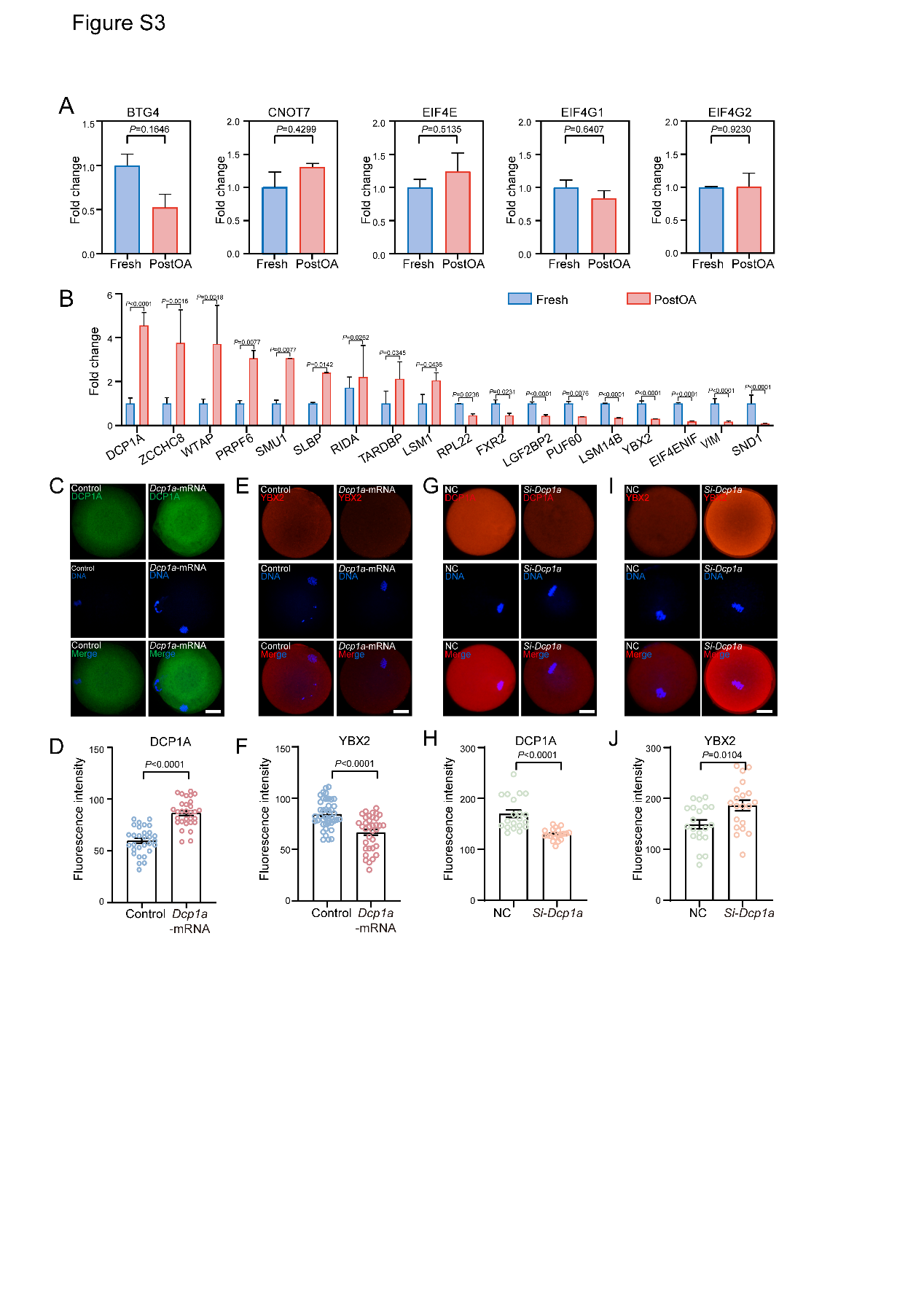


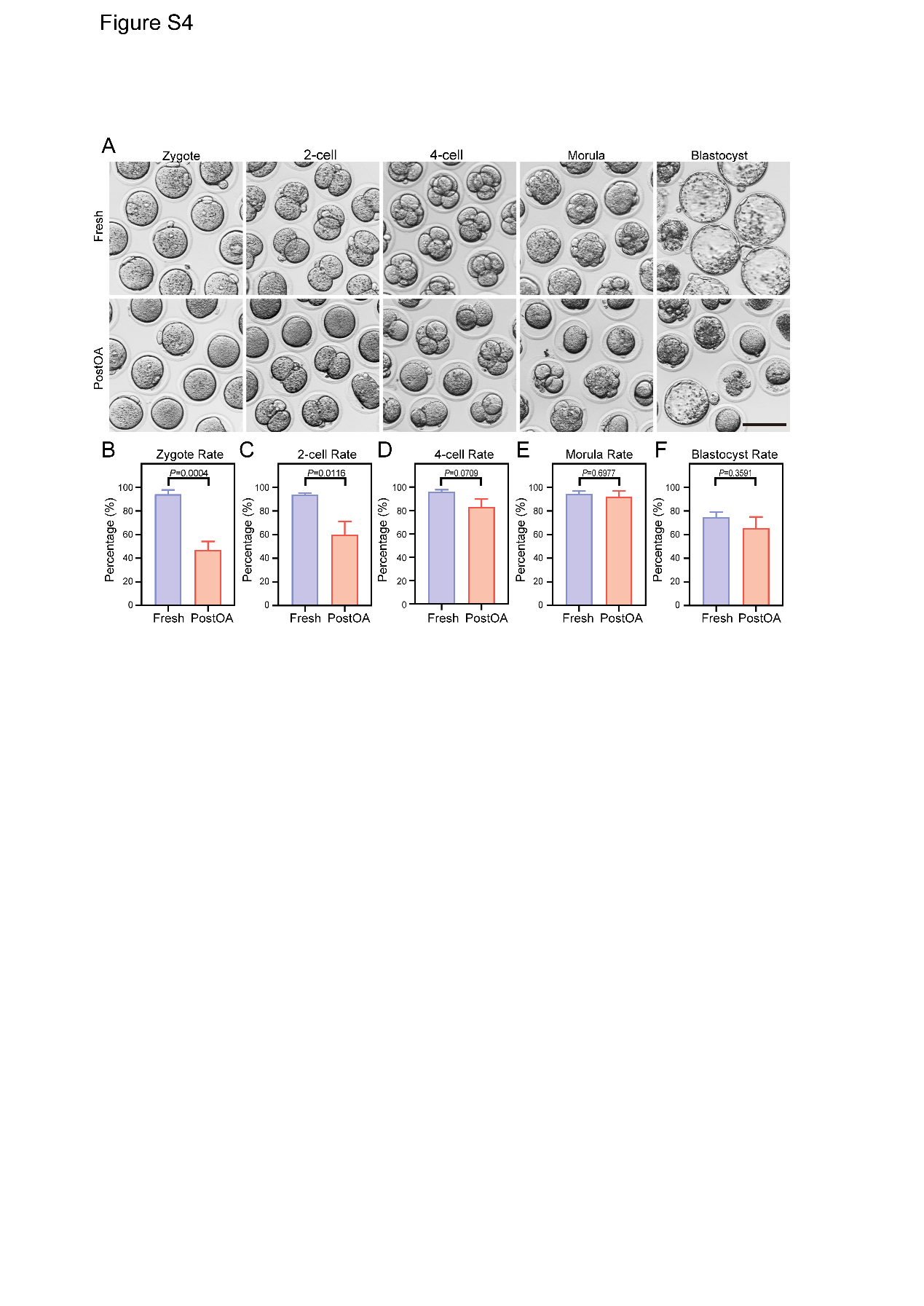


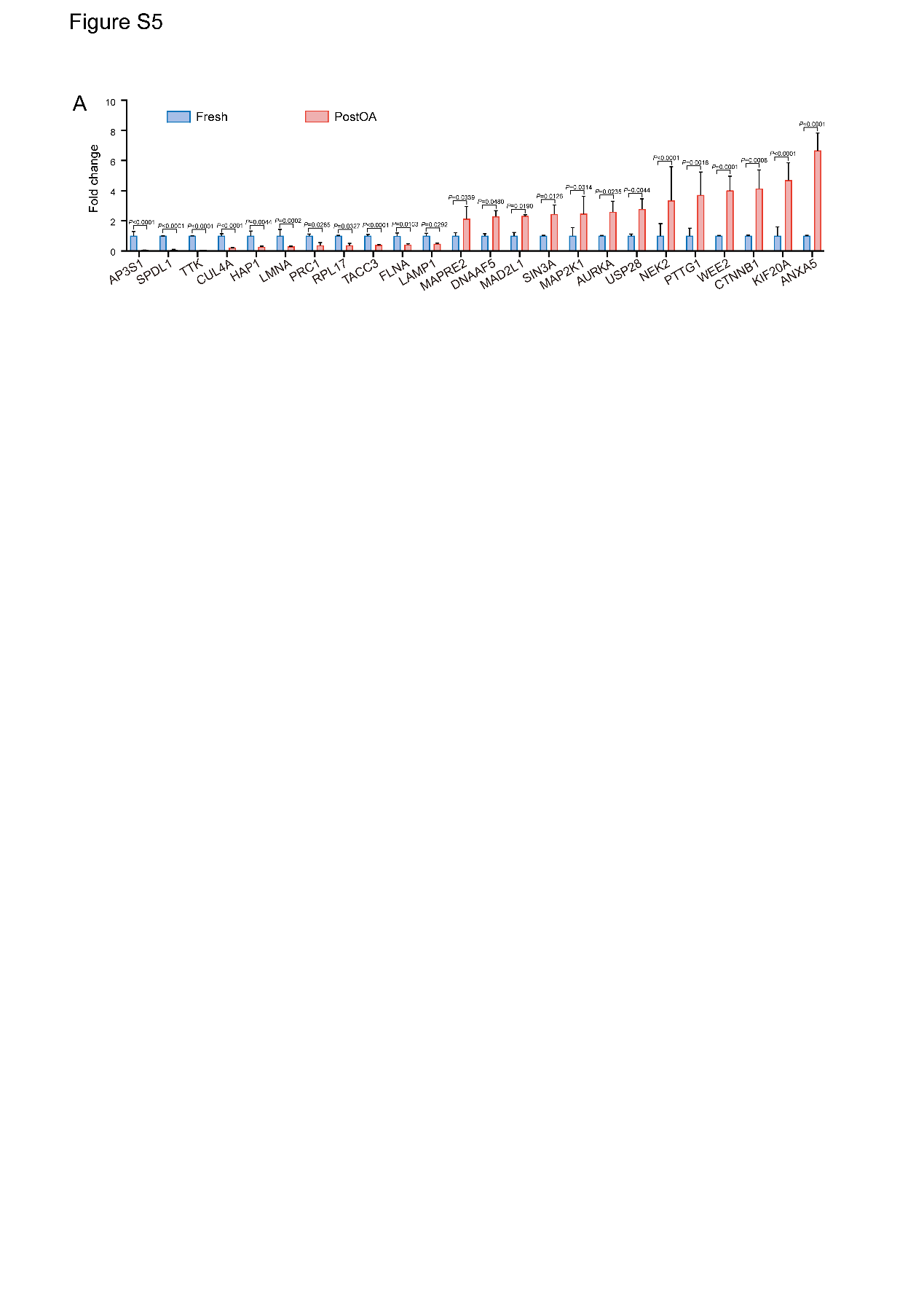

Supplement: Supplementary file 1 — Figure S1 [file CPR-58-e13766-s003.docx]
