# Supplementary material for: Multi‐omics revealed that DCP1A and SPDL1 determine embryogenesis defects in postovulatory ageing oocytes
Source: Cell Prolif. 2024 Dec 4;58(3):e13766. doi: 10.1111/cpr.13766 (PMC11882766; doi:10.1111/cpr.13766)
Supplement: Supplementary file 4 — Table S3 [file CPR-58-e13766-s001.docx]

**Table S3. Antibodies used in this paper.**

| **Primary antibodies** | **Vendor** | | **Dilution** | Source |
| --- | --- | --- | --- | --- |
| DCP1A | Affinity(AF0551) | | 1:200(IF)  1:1000(WB) | Rabbit |
| YBX2 | Affinity(DF3482) | | 1:200(IF)  1:1000(WB) | Rabbit |
| SPDL1 | Bioss(bs-2321R) | | 1:200(IF)  1:1000(WB) | Rabbit |
| TTK | Abclonal(A2500) | | 1:200(IF)  1:1000(WB) | Rabbit |
| α-Tubulin | Sigma(T7451) | | 1:1000(IF) | Mouse |
| Gapdh(WB) | Immunoway(YM3040) | | 1:1000 | Mouse |
| **Secondary antibodies**  CyTM-3-conjugated donkey anti-rabbit (IF)  FITC-conjugated donkey anti-rabbit (IF)  HRP-conjugated goat anti- Mouse IgG (WB)  HRP-conjugated goat anti- rabbit IgG (WB) | | Jackson (711-165-152)  Jackson (711-095-152)  Thermofisher (31430)  Thermofisher (31460) | 1:200  1:200  1:1000  1:1000 | Donkey  Donkey  Goat  Goat |

**Table S4. Primers Used for Quantitative RT-PCR.**

| **Genes** | **Forward primer sequence** | **Reverse primer sequence** |
| --- | --- | --- |
| *Zar1* | AGAGCGCCTATGTGTGGTGT | TCTCCCACACAAGTCTTGCC |
| *Tle6* | ATCCAGTCGGTATTTGTCCATCG | AGGTCTGGGGTTCTACTGAAG |
| *Tet3* | TGCGATTGTGTCGAACAAATAGT | TCCATACCGATCCTCCATGAG |
| *Mos* | GGGAACAGGTATGTCTGATGCA | CACCGTGGTAAGTAAGTGGCTTTATACA |
| *Gdf9* | TCTTAGTAGCCTTAGCTCTCAGG | TGTCAGTCCCATCTACAGGCA |
| *Dnmt1* | AAGAATGGTGTTGTCTACCGAC | CATCCAGGTTGCTCCCCTTG |
| *Bmp15* | TCCTTGCTGACGACCCTACAT | TACCTCAGGGGATAGCCTTGG |
| *Dcp1a* | ACAACACGACCCCTATATCACC | CGGTTGACAATGGTAAAGCCA |
| *Spdl1* | GGTGCAGCTACATAGGAGCC | TCTTCTGACTTGTGAGCGAGA |
| *Gapdh* | GTCATTGAGAGCAATGCCAG | GTGTTGCTACCCCCAATGTG |
| *NC* | UUCUCCGAACGUGUCACGUTT | ACGUGACACGUUCGGAGAATT |
| *SiDcp1a-1* | GGUUGAGCCAGUGAAUAAATT | UUUAUUCACUGGCUCAACCTT |
| *SiDcp1a-2* | GGGAGAUGCAUCACAGAAATT | UUUCUGUGAUGCAUCUCCCTT |
| *SiDcp1a-3* | CCCACAGCAUGACCAAAUATT | UAUUUGGUCAUGCUGUGGGTT |
